# Discovery of Synergistic Drug Combinations for Colorectal Cancer Driven by Tumor Barcode Derived from Metabolomics “Big Data”

**DOI:** 10.3390/metabo12060494

**Published:** 2022-05-30

**Authors:** Bo Lv, Ruijie Xu, Xinrui Xing, Chuyao Liao, Zunjian Zhang, Pei Zhang, Fengguo Xu

**Affiliations:** 1 Key Laboratory of Drug Quality Control and Pharmacovigilance, China Pharmaceutical University, Nanjing 210009, China; 1831010037@stu.cpu.edu.cn (B.L.); 3219010180@stu.cpu.edu.cn (R.X.); 3120070217@stu.cpu.edu.cn (X.X.); 1821010176@stu.cpu.edu.cn (C.L.); zunjianzhangcpu@hotmail.com (Z.Z.); 2 State Key Laboratory of Natural Medicine, China Pharmaceutical University, Nanjing 210009, China

**Keywords:** tumor barcode, Bayesian vote-counting, colorectal cancer, metabolomics, drug combination, Irinotecan

## Abstract

The accumulation of cancer metabolomics data in the past decade provides exceptional opportunities for deeper investigations into cancer metabolism. However, integrating a large amount of heterogeneous metabolomics data to draw a full picture of the metabolic reprogramming and to discover oncometabolites of certain cancers remains challenging. In this study, a tumor barcode constructed based upon existing metabolomics “big data” using the Bayesian vote-counting method is proposed to identify oncometabolites in colorectal cancer (CRC). Specifically, a panel of oncometabolites of CRC was generated from 39 clinical studies with 3202 blood samples (1332 CRC vs. 1870 controls) and 990 tissue samples (495 CRC vs. 495 controls). Next, an oncometabolite-protein network was constructed by combining the tumor barcode and its involved proteins/enzymes. The effect of anti-cancer drugs or drug combinations was then mapped into this network by the random walk with restart process. Utilizing this network, potential Irinotecan (CPT-11)-sensitizing agents for CRC treatment were discovered by random forest and Xgboost. Finally, a compound named MK-2206 was highlighted and its synergy with CPT-11 was validated on two CRC cell lines. To summarize, we demonstrate in the present study that the metabolomics “big data”-based tumor barcodes and the subsequent network analyses are potentially useful for drug combination discovery or drug repositioning.

## 1. Introduction

Cancers are known as metabolic diseases that are characterized by immoderate proliferation. To meet the nutrient requirements for the growth of tumors, alterations in various metabolism such as amino acid, carbohydrate, and nucleic acid pathways often occur [1,2,3]. Such alterations are specific to tumor types [4], indicating that the metabolic alteration profile of a certain cancer may be considered as its identification barcode or an I.D. card. This tumor barcode has enormous potential in disease diagnosis and anti-cancer drug discovery as it is representative of the metabolic phenotype. This requires the oncometabolites included in a tumor barcode should be as stable and comprehensive as possible. Large clinical cohort studies are ideal for obtaining reliable oncometabolites as potential cofounding factors or individual differences are better controlled than small studies. However, substantial time and financial investments are needed to conduct such studies.

An alternative to large cohort studies is integrating the invaluable existing data from small metabolomics studies. First, the oncometabolite coverage would be significantly increased by combining multiple small studies as different analytical platforms are often applied in these studies. Further, the oncometabolites reported to regulate consistently in studies from different laboratories are more reliable than the ones only in a few studies. More importantly, there has been a substantial accumulation of metabolomics data in this field in the past decade, which could provide enormous information on the metabolic alterations in various types of cancers. The integration of those data would make it possible to establish the tumor identification barcodes. However, due to different experimental conditions applied in the individual laboratory (e.g., instrument, analytical method [5], and pretreatment of samples [6]), metabolomics studies of identical diseases could lead to distinct results, making it difficult to obtain comparable information from these small-scale studies of various sources. Therefore, a strategy to extract homogeneous information from individual heterogeneous studies to create identification barcodes in cancers is highly needed.

There are mainly two types of frequently used data integration methods: semi-quantitative (e.g., vote-counting method) and quantitative (directly synthesizing quantitative effect size such as fold-change and odd ratio). Due to the heterogeneity of these quantitative effect sizes [7,8,9,10], the vote-counting method is more suitable for metabolomics data integration and has been successfully used to extract biomarkers according to the robustness cross-published studies by sign test [11,12,13]. Recently, it was also introduced in meta-analyses of cancer metabolomics studies [13,14]. As mentioned above, a tumor barcode should be specific to tumor types; therefore, it is more appropriate to integrate data from only one tumor type instead of multiple cancers. In this case, the number of metabolomics studies that mention the same metabolite(s) might be insufficient (often <6), which would significantly weaken the efficiency of sign tests used in classical vote-counting strategies that usually require much larger replicates. Compared to traditional frequentists statistics frames (such as the sign test in the vote-counting method) in which high statistic power often relies on a large sample size, the Bayesian frame integrates information of each observation into posterior distribution, which has been proven more efficient for the integration of small sample size studies [15].

Therefore, we propose an integrated vote-counting and Bayesian frame strategy (called the Bayesian vote-counting method) to combine existing metabolomics studies and establish tumor barcodes, as a proof of concept, for colorectal cancer (CRC). A comprehensive network consisting of the oncometabolites in the barcode, as well as interactive enzymes, translators, or other proteins obtained from open-source databases such as Stitch [16] and String [17], was constructed. This network can be regarded as a space to span the information on these interactions. The effect of anticancer drugs or drug combinations was mapped into this space by the random walk with restart method [18]. It is known that Irinotecan (CPT-11) is a first-line anticancer drug in clinical for the treatment of CRC with an unsatisfactory effective rate of <50% [19]. In the present study, utilizing the established tumor barcodes, constructed network, as well as the Drugs Combination Database (DCDB) [20], drugs/compounds that can sensitize CPT-11 were discovered. In the end, a compound named MK2206 was highlighted, and its synergy with CPT-11 was validated on two CRC cell lines, indicating that the concept of utilizing metabolic information for the discovery of anticancer drug combinations has a great potential. Compared to experimental screening methods, this strategy is much more time and cost effective. More importantly, the established workflow can be readily applied to other cancer types or diseases.

## 2. Results and Discussion

### 2.1. Study Workflow

**Figure 1:**
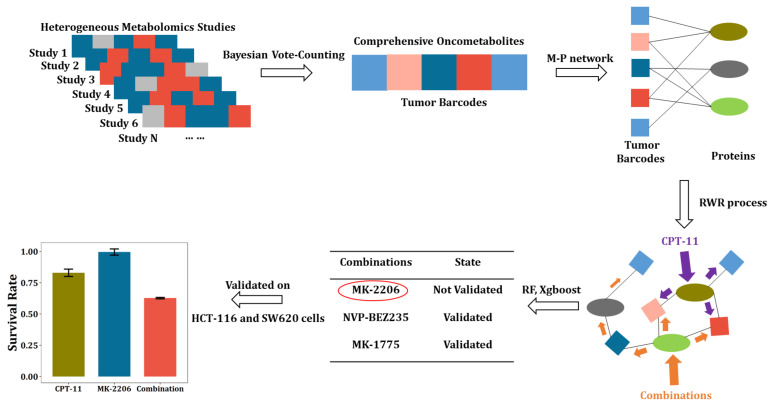
Workflow of tumor barcode establishment and its application in discovery of anti-cancer drug combinations.

The overall workflow of this study is presented in Figure 1. First, the existing literature involving CRC metabolomics was searched and curated, and the information on oncometabolites, including names, regulation directions, sample types, analytical methods, etc., was extracted to construct the literature library. Based on the library, the tumor barcodes were constructed by the Bayesian vote-counting method, of which advantages over the sign test under small sample size conditions had been validated by simulation. Then, utilizing the tissue tumor barcode, we constructed an oncometabolite-protein network. The effect of anti-cancer drugs or drug combinations were then mapped into this network by the random walk with restart process. Next, the random forest and Xgboost were applied for the prediction of synergistic CPT-11-based drug combinations. Eventually, MK-2206 was found to be a potent sensitizing agent of CPT-11 and their synergy was validated on two CRC cell lines including HCT-116 and SW-620.

This workflow utilizes published small-scale metabolomics studies to generate disease barcodes that are highly specific. The barcodes and their extended metabolite–protein network can be used for disease diagnosis or therapeutics finding, etc. In our study, we initially intended to use this network to facilitate the discovery of novel antitumor targets. However, this unsupervised method resulted in very regular targets, interfering of which led to ineffectiveness. Combination therapy has been shown effective in treating complex chronic diseases such as cancer, and has become an indispensable strategy to overcome resistance in anticancer drugs. Therefore, we creatively combined the CRC metabolite–protein network with an anticancer drug combination database. This supervised strategy greatly improved the probability of obtaining meaningful drug combinations.

### 2.2. Literature Searching

**Figure 2:**
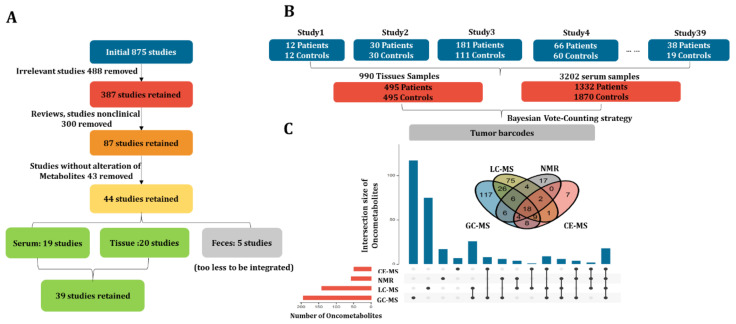
CRC metabolomics data collection. (**A**) The enrollment criteria of CRC metabolomics studies; (**B**) structure of the final dataset including subject number, sample size, and sample type; (**C**) Venn plot and upset plot for visualizing the metabolite coverage of different instrument platforms including GC-MS, LC-MS, NMR, and CE-MS.

Initially, 875 articles involving CRC metabolomics published between 2000~2020 were collected. As shown in Figure 2A, 488 publications irrelevant to CRC metabolomics, 300 review articles, and non-clinical studies were also removed post a closer check of the contents. Of the remaining 87 papers, 43 were further excluded due to missing information on metabolite changing trends. As a result, 44 studies were obtained, 19 of which were conducted on blood, 20 on tissue, and the remaining 5 on feces. The fecal studies were also removed due to the very small number. In the remaining 39 studies, the sample size of blood and tissue was 3202 (1332 CRC vs. 1870 control) and 990 (495 CRC vs. 495 control), respectively (Figure 2B). On the other hand, four different types of analytical platforms including GC-MS (gas chromatography–mass spectrometry), LC-MS (liquid chromatography–mass spectrometry), NMR (nuclear magnetic resonance), and CE-MS (capillary electrophoresis–mass spectrometry) were applied either separately or as a combination. Collectively, 330 metabolite biomarkers were reported (Figure 2C). First, the combined sample size from multiple small studies resembles large cohort studies. Second, the detection range of oncometabolites was enhanced by combining results from different analytical platforms. More importantly, the reliability of oncometabolites was also enhanced by mutual validation.

### 2.3. Comparisons between Bayesian Frame and Sign Test

**Figure 3:**
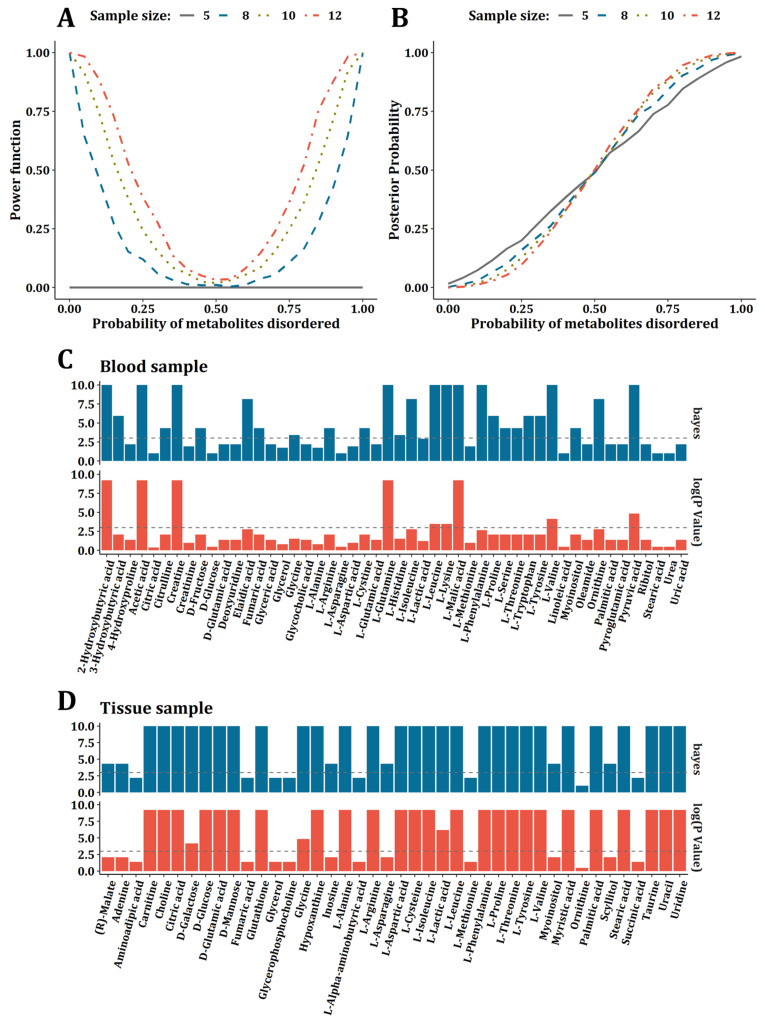
Comparisons between Bayesian frame and sign test. (**A**) The power of sign test under the sample size of 5, 8, 10, and 12. (**B**) The relationship between expectation of posterior probability and real probability used to generate simulation data under the sample size of 5, 8, 10, and 12. (**C**) Bar plot for comparison between oncometabolites detected by Bayesian frame and sign test for blood samples. (**D**) Bar plot for comparison between oncometabolites detected by Bayesian frame and sign test for tissue samples.

To compare the performance of the Bayesian frame and sign test, we conducted a simulation test. As a result, the statistic power of the sign test was improved as a sample size increasing under identity disorder probability; however, while the sample size was less than 8, the statistic power was too low and no significant oncometabolites could be detected while the sample size was <5 (Figure 3A). Due to the frequency of oncometabolites being mostly <8 in the dataset that we collected, the sign test in the vote-counting method was underpowered for the data integration. In the case of the Bayesian frame, it was found that the expectation of posterior probability of oncometabolite disorders was close to the real probability used to generate simulated samples. Further, the fitness between the expectation of posterior probability and real disorder probability was robust even when the sample size was equal to 5 (Figure 3B). Therefore, we concluded that the Bayesian frame was more suitable for integrating metabolomics studies than the sign test. On the other hand, with the criteria of the absolute Bayesian factor >3 or *p*-value < 0.05 in the sign test, 27 oncometabolites were detected by the Bayesian method, while only 9 by sign test from blood samples (Figure 3C). In tissue samples, it was 34 (Bayesian) vs. 28 (sign test) (Figure 3D).

### 2.4. CRC Tumor Barcodes Establishment

Based on the 39 studies and 330 potential biomarkers that we collected, the vote-counting method was utilized to integrate these studies with a frequency (number of studies reporting up- or downregulation) ≥3. As a result, a total of 254 metabolites were included for further analysis. The Bayesian statistical frame was used to evaluate the reliability of the metabolite alterations. Every potential oncometabolite was evaluated based on more than 200 blood samples (100 CRC vs. 100 controls) or 60 tissue samples (30 CRC vs. 30 controls). In the end, 50 blood and 43 tissue oncometabolites with a frequency ≥3 were focused on, respectively. The frequency, cumulative sample size, and the Bayesian factor of the oncometabolites from blood and tissue samples are listed in Tables S1 and S2, respectively.

**Figure 4:**
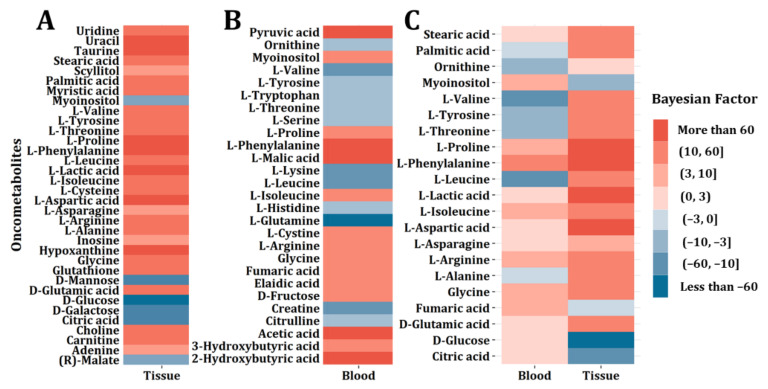
CRC tumor barcode construction. (**A**) Tumor barcode composed of significantly altered oncometabolites for CRC tissue samples. (**B**) Tumor barcode composed of significantly altered oncometabolites for CRC blood samples. (**C**) Tumor barcodes composed of oncometabolites both appearing in blood and tissue samples and showing significant alterations.

We then constructed the barcodes with significantly altered oncometabolites (the absolute Bayesian Factor >3). Among the 43 oncometabolites found in tissue, 28 significantly upregulated (Bayesian Factor >3) and 6 downregulated (Bayesian Factor <−3) were used (Figure 4A). In the 50 blood oncometabolites, 15 upregulated (Bayesian Factor >3) and 12 downregulated (Bayesian Factor <−3) were included (Figure 4B). Moreover, 21 oncometabolites were found significantly altered in blood or tissue (Figure 4C). The colors in Figure 4A,B are deeper because the absolute Bayesian factors are all larger than 3. Figure 4C represents a tumor barcode consisting of common oncometabolites appearing in both blood and tissue (Frequency >3) with significant alteration in at least one type of sample rather than both. In other words, not all the absolute Bayesian factors are larger than 3 in Figure 4C. Thus, the colors in Figure 4C seem to be lighter than those in Figure 4A,B.

As we can see, the regulation directions in the two different biological species were not the same. Oncometabolites including isoleucine, arginine, cysteine, and proline were upregulated both in blood and tissue, while stearic acid, palmitic acid, lactic acid, and aspartic acid only showed significant regulations in tissue, and ornithine and glutamine only showed significant regulations in blood. Moreover, the change directions of valine, tyrosine, threonine, phenylalanine, leucine, asparagine, alanine, glycine, fumaric acid, glutamic acid, glucose, and citric acid are opposite in blood and tissue.

Such differences in metabolic profile changes among distinct sample types have been found in cancer including CRC [21,22], which might be caused by transporters, disruption, of homeostasis, enzymes, etc. The transporters can be perceived as bridges between blood and tissues, which can play a role in causing the metabolic difference. For example, it has been reported that SCL1A5 (Solute Carrier family 1, Member 5), a glutamine transporter, was upregulated in CRC [23,24]. Thus, a large amount of glutamine was transported into tumor cells as a carbon source for many biosynthetic pathways, leading to the downregulation of glutamine in blood. Meanwhile, glutamine was consumed largely after being transported into tumor cells, resulting in a stable concentration in CRC tissues. Enzymes and transcription factors might be important factors that could influence oncometabolites regulation as well. For example [25], c-MYC (MYC proto-oncogene), a key regulator in cancers, could markedly inhibit the expression of POX/PRODH (POX, peroxidase; PRODH, proline dehydrogenase 1, mitochondrial) to further promote the biosynthesis of proline. Thus, proline showed a noticeable upregulation both in blood and tissue samples. Further investigations are needed to understand the reasons that lead to the differences between blood and tissues. The regulation mechanisms will become more understood along with the gradual advances in the field. Future findings might result in new drug targets that can be used for discovering novel anticancer treatments.

The current tumor barcode is only constructed with non-lipids. The reason is that the unambiguous identification of lipid species presents as well as inaccurate quantitation remains a significant challenge. For example, studies [26,27] reported detailed structure information of lipids such as LPC(18:2(9Z,12Z)) and LPC(18:1(9Z)) in CRC. However, other studies [8,28] presented these lipids as LPC(18:2) and LPC(18:1). Further development in lipid identification might largely expand the tumor barcode in the future and a more standardized report of the lipids would benefit the data integration.

### 2.5. Oncometabolite-Protein Network Construction and Its Application in the Discovery of Potential Drug Combinations

Considering that the oncometabolites produced by cancer cells located in the tumor are more closely related to disease pathological mechanism than those in blood that could be a merged effect from all the organs, we used 43 tissue oncometabolites to build the oncometabolite-protein network (Figure S1 in Supplementary Materials). This network presents 804 nodes and 9403 interactions, and the ultimate predictor matrix consisted of 541 rows and 804 columns. The rows represent the drugs combinations in the DCBD database [20] treated on CRC cells including SW-620, HT29, HCT116, COLO320DM, LD1, HCT15, LOVO, RKO, and SW837. The columns represent nodes in the network.

The parameter “mtry” in the random forest (Figure S2, Supplementary Materials), “max_depth” and “nrounds” in the Xgboost were selected (Figure S3, Supplementary Materials) when the cross-validation errors were the lowest. The performance of two major ensemble learning methods was shown in Table S3 (Supplementary Materials). No significant differences between these two methods were found. As a result, potential sensitizing agents of Irinotecan (CPT-11) were highlighted including MK-2206, NVP-BEZ235, MK-1775, and Raloxifene. Among them, the potential synergic effect with CPT-11 of MK-2206, NVP-BEZ235, and MK-1775 were supported by both random forest and Xgboost. The synergism of NVP-BEZ235 and MK-1775 with CPT-11 on CRC cells has been reported elsewhere [29,30,31]. Therefore, in the present study, we chose to further validate the synergistic effect of MK-2206 and CPT-11 on CRC cell lines.

### 2.6. Validation for the Synergistic Effect between MK-2206 and CPT-11 on CRC Cells

The synergistic effect of MK-2206 as a potential sensitizing agent of CPT-11 needs to be validated. In this study, in vitro experiments on two different CRC cell lines were performed for this purpose. Referring to existing literature, the combination index Q [32] and Bliss independent model [33] were applied for the evaluation of the synergism.

**Figure 5:**
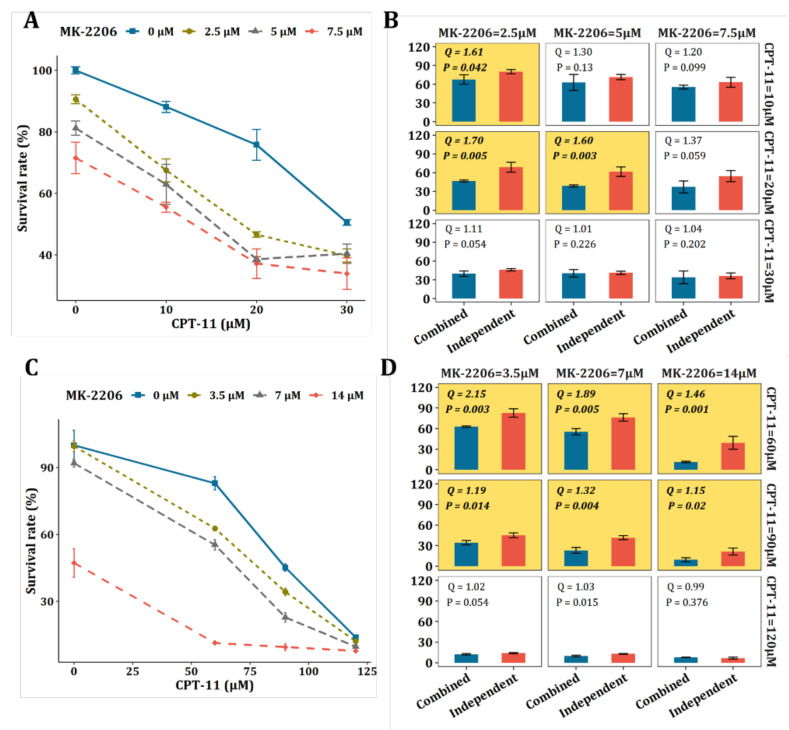
Validation of the synergy between MK-2206 and CTP-11 on CRC cell lines. (**A**) Proliferation assay for CPT-11, MK-2206, and their combination on HCT-116 cells at 48 h. (**B**) Bliss independence model for the evaluation of the synergy between CPT-11 and MK-2206 on HCT-116 cells. The yellow background represents significant synergisms with synergy Q index >1.15 and *p*-value < 0.05. (**C**) 48 h proliferation assay (MTT) for CPT-11, MK-2206, and their combination on the SW-620 cell line. (**D**) Bliss independence model for the evaluation of the synergy between CPT-11 and MK-2206 on SW-620 cells. The yellow background represents significant synergisms with synergy Q index ≥ 1.15 and *p*-value < 0.05.

The IC_50_ of CPT-11 was determined on HCT-116 and SW-620, respectively (Figure S5) and the concentrations of CPT-11 were set accordingly. Figure 5A,B show the results from HCT116 cells. As shown in Figure 5A, the effect of CPT-11 or MK-2206 was both dose-dependent. More importantly, MK-2206 increased the sensitivity of CPT-11 at all designed concentration levels. We calculated the combination index Q as well as the *p*-value of the Bliss independence model and the results are shown in Figure 5B. Significant synergism between CPT-11 and MK-2206 was observed on HCT-116 cells when (1) MK-2206 = 2.5 μM, CPT-11 = 10 μM; (2) MK-2206 = 2.5 μM, CPT-11 = 20 μM; and (3) MK-2206 = 5 μM, CPT-11 = 20 μM. Likewise, this synergistic effect was validated on another CRC cell line, i.e., SW620. As we can see from Figure 5C, the effect of CPT-11 or MK-2206 on SW620 cells was also dose-dependent and MK-2206 increased the sensitivity of SW-620 to CPT-11 at all designed concentration levels. Significant synergism between CPT-11 and MK-2206 can be seen on SW-620 cells when (1) MK-2206 = 3.5 μM, CPT-11 = 60 μM; (2) MK-2206 = 3.5 μM, CPT-11 = 90 μM; (3) MK-2206 = 3.5 μM, CPT-11 = 120 μM; (4) MK-2206 = 7 μM, CPT-11 = 60 μM; (5) MK-2206 = 7 μM, CPT-11 = 90 μM; and (6) MK-2206 = 7 μM, CPT-11 = 120 μM (Figure 5D).

It was found that SN-38, a metabolic product of CPT-11, can activate Akt kinase, which could lead to proliferation of tumor cells [24]. The active Akt kinase could impair the inhibitory effect of CPT-11 on tumor cells. On the other hand, the MK-2206 is a selective inhibitor of Akt1/2, which can inhibit the activating effect of SN-38. This might explain the significant synergism between CPT-11 and MK-2206 observed in our study. However, it needs deeper investigations. Overall, the synergistic effect of MK-2206 with CPT-11 was validated on two different CRC cell lines, demonstrating that the strategy of tumor barcode and the oncometabolite-protein network have a great potential in finding novel drug combinations.

Colorectal cancer can be divided into hPMS1, hPMS2, hMSH3, hMSH6, hMLH3, and EXO1 according to the human DNA MMR system [34] and different molecular cancer types may have distinct metabolic patterns. Ideally, a tumor barcode should be constructed for each molecular subtype, based on which more precise treatment can be discovered. However, most of the existing metabolomics studies failed to provide detailed disease information. More comprehensive information on enrolled cases in metabolomics studies would largely promote precise medicine in the future.

## 3. Materials and Methods

### 3.1. Data Collection

The workflow of constructing tumor identification barcodes is illustrated in the case of CRC. First, we conducted a systematic literature search regarding CRC metabolomics studies published between 2000 and 2020 on the Web of Science (https://www.webofscience.com/wos/alldb/basic-search, accessed on 1 October 2020). Our search terms consisted of methodology types (metabolomics, metabonomics, metabolic profiling) and study objects (colon cancer, colonic neoplasms, and colorectal cancer) in human samples.

The obtained literature received a closer check on title and abstract. The duplicated and irrelevant ones were not included. Review articles or original articles without metabolite annotations were also removed. Further, articles that only report the significantly changed metabolites without mentioning the changing trends were also removed. All the reserved studies were used to generate a data set and labeled by sample types (i.e., serum, cancer tissue, or feces). Information including methodological parameters (sample type, instrument, method, etc.) and reported metabolites (concentration, fold-change, up- or downregulation, etc.) were then extracted.

### 3.2. Tumor Barcodes Establishment

In some publications, the reported metabolites might be annotated with ambiguous names or different identifications (e.g., CAS No., HMDB ID, InChIkey), posing challenges in further information processing. Therefore, all the extracted metabolites were standardized with KEGG ID referring to the KEGG database (http://www.kegg.jp/, last search on 20 February 2021).

In the vote-counting method, the frequency of a metabolite was defined as the number of times that this metabolite was reported up- or downregulated in the data set generated as described in Section 3.1. The alterations were considered significant if reported in ≥3 independent studies.

Oncometabolites upregulating (denoted by “+1”) or downregulating (denoted by “−1”) in CRC patients were extracted. If the count of “+1” was higher than “−1”, success was determined as upregulation and vice versa. In the Bayesian vote-counting strategy, we hypothesized that the prior probability of success (devoted by *θ*) was uniformly distributed as Unif(0,1) because there is no effective prior information. According to the Bayesian theory, the posterior distribution can be represented by the following formula.

$$P\left(\theta |X\right)=\frac{P\left(X|\theta \right)P\left(\theta \right)}{\int_{\theta }^{\;}P\left(X|\theta \right)P\left(\theta \right)}$$

where $P\left(\theta |X\right)$ represents the posterior distribution of success probabilities (up- or downregulation of an oncometabolite); and $P\left(\theta \right)$ represents the prior distribution of probabilities. $P\left(X|\theta \right)\;$ represents the conditional likelihood under a given θ. Finally, $P\left(\theta |X\right)$ can be derived from $Beta\left(x+1,n-x+1\right)$ where x is the number of successes, and n is the total number of studies mentioning the given oncometabolite [35]. The Bayesian factor was calculated as the ratio of the posterior probability of metabolites alteration conforming to the null hypothesis ($P\left(\theta >0.5|X\right)$) and unconforming to the null hypothesis ($P\left(\theta <0.5|X\right)$). In this study, the regulation was considered significant when the absolute Bayesian factors >3 refer to existing literature [36]. Consequently, the oncometabolites with absolute Bayesian factors >3 were used to construct the tumor barcodes. Each bar represents one oncometabolite, the colors of bars represented up- or downregulation of the metabolite, and the gradients of colors from scaled Bayesian factors represented the reliability of this metabolite alteration in CRC.

### 3.3. Oncometabolite–Protein Network Construction with Random Walk with Restart

The tissue tumor barcode consisting of oncometabolites can be considered as seeds of a network that can be expanded by integrating with enzymes, translators, and other functional proteins that are potentially involved in metabolism reprogramming. In this study, the oncometabolite–protein network was constructed based on the tissue barcode as it is more relevant to the metabolic reprogramming of CRC. Interactions between proteins and each oncometabolite were gathered from the Stitch database (http://stitch.embl.de/, accessed on 1 March 2021) as individual networks of oncometabolites. These individual networks of oncometabolites were aggregated into a global network by protein–protein interactions after isolated parts were removed from the String database (https://string-db.org/, accessed on 1 March 2021). This oncometabolite–protein network provided a space into which the effects of drug combinations can be mapped. The process includes four steps: Step1. For a walker on the oncometabolite–protein network, the transition probability is defined as:

$$W\left(i,j\right)=\frac{A\left(i,j\right)}{\sum_{k=1}^{N}A\left(i,k\right)}$$

where *N* is the number of nodes in the oncometabolite-protein network.

Step2. Define the start as the protein targets affected by drugs or drug combinations to simulate the effects of these drugs, which is noted as $\;P_{0}$.

Step3. Define a restarting parameter λ which represents the probability that the walker on network returns the start. The steady state of the random walk with restart process can be derived as $P_{t}=\left(1-\lambda \right)W^{T}P_{t-1}+\lambda P_{0}$. The formula can be further transformed into $P_{t}=\left(1-\lambda \right){\left(I-\lambda W^{T}\right)}^{-1}P_{0}$ according to the steady state where $\;P_{t}=P_{t-1}$. The λ was determined as 0.5 arbitrarily or by cross-validation.

Step4. Based on the oncometabolite–protein network and random walk with restart process, the effects of drug combinations can be mapped into the numerical space with N dimensions, which can be used as predictors to predict whether a drug combination is synergistic.

### 3.4. Prediction Models for Finding Potential Sensitizing Agents of Irinotecan

Based on numerical space created by oncometabolite–protein network and random walk with restart process, ensemble learning models including random forest and Xgboost were constructed. The dataset was divided into the training set (80%) and the test set (20%). A 10-fold cross-validation was used to tune parameters and estimate the performance on model fitness and accuracy on the test set was used to estimate the performance of prediction.

### 3.5. Cell Experiments

Cell lines: Human colon carcinoma cell lines HCT116 and SW620 were purchased from Nanjing Hongxin Biotechnology Co., Ltd. (Nanjing, China). These two cell lines were identified by STR analysis prior to the experiments. Cells were cultured in high glucose Dulbecco’s modified Eagle’s medium (DMEM, Grand Island, NY, MA, USA) supplemented with 10% fetal bovine serum FBS (*v*/*v*) (Gibco, Grand Island, NY, USA) and 1% penicillin (100 units/mL)/streptomycin (100 µg/mL) (Hyclone, Logan, UT, USA). Cultures were maintained in a humidified incubator at 37 °C in 5% CO_2_.

Regents: MK-2206 di-hydrochloride (CAS 1032350-13-2) was purchased from GLPBIO Co., Ltd. (Shanghai, China). CPT-11 (CAS 97682-44-5) was purchased from Aladdin Bio-Chem Technology Co., Ltd. (Shanghai, China). Dosage setting of drugs and their combination for HCT-116 and SW-620 cell lines are shown in the Result and Discussion part. All drugs were diluted by culture medium while used.

MTT assays: Cells were seeded into a 96-well plate at 6 × 10^3^ cells/100μL medium/well and were incubated overnight. After the vehicle and or drug treatment for 48 h, 20 μL/well of MTT (5 mg/mL) (3-(4, 5-dimethylthiazol-2-yl)-2, 5-diphenyltetrazolium bromide, BEYOTIME, China) was added and incubated for 4 h at 37 °C. Then, DMSO was added to dissolve the formazan crystals, and the optical density (OD) was measured at 490 nm by a microplate reader (Tecan, Switzerland). The survival rate and inhibitory rate was calculated according to the following equations:

$$\mathrm{Survival}\;\mathrm{rate}\left(\%\right)=\left(1-\frac{OD_{Treatment}}{OD_{Blank}}\right)\times 100\%\;\mathrm{Inhibitory}\;\mathrm{rate}\left(\%\right)=1-\;\mathrm{Survival}\;\mathrm{rate}$$

### 3.6. Data Analysis

The synergism between CPT-11 and MK-2206 was evaluated by combination index Q [34] and the Bliss independence model. The combination index Q was calculated by the following formula.

$$\mathrm{Combination}\;\mathrm{index}\;Q=\frac{E_{Combination}}{E_{CPT-11}+E_{MK-2206}-E_{CPT-11}\times E_{MK-2206}}$$

where *E* represents inhibitory rate.

The t-statistic of Bliss independence model was calculated as:

$$t=\left(\frac{\left(y_{1}+y_{2}-y_{3}\right)\sqrt{\sum_{i=1}^{3}n_{i}-3}}{\sqrt{\sum_{i=1}^{3}\sum_{j=1}^{n_{i}}\left(y_{ij}-{\bar{y}}_{i}\right)\sum_{i}^{3}n_{i}^{-1}}}\right)\times 100\%$$

Where *y* represents the log survival rate for each group, and *n* represent the number of replications for each group.

A two-side *p*-value of t-statistic with degree of freedom $\sqrt{\overset{3}{\underset{i=1}{\sum }}n_{i}-3}$ was calculated. In our study, a combination index Q ≥ 1.15 and *p*-value < 0.05 in the Bliss independence model was considered as significant.

## 4. Conclusions

In this study, to construct tumor barcodes consisting of more reliable and comprehensive oncometabolites from heterogeneous metabolomics “big data”, the Bayesian vote-counting strategy was proposed. This strategy enables not only the great expansion of sample size, but also the extension of the metabolite coverage. Moreover, to establish a connection between the metabolic alterations and the discovery or repositioning of drugs, an oncometabolite–protein network was constructed. As a proof of concept, we demonstrated here the value of this network in finding the potential sensitizing agent of CPT-11 for the inhibition of CRC cell proliferation. At last, MK-2206 was discovered and the synergism between MK-2206 and CPT-11 was validated on HCT-116 and SW-620 cells. Limitations of the current study include: (1) a more systemic search following the PRISMA statement might enhance the robustness and reproducibility of the results; (2) only non-lipids are included for the barcode construction due to the unambiguous identification of lipid species and inaccurate quantification; and (3) the molecular subtype of CRC was not considered with limited information recorded in the existing metabolomics studies. Nevertheless, our study revived the vote-counting method for the integration of metabolomics studies in a small sample size situation by combining the Bayesian frame. More importantly, the proposed workflow provides a creative route for the discovery of drug combinations from published metabolomics studies. As this strategy is dependent on the quality and quantity of published metabolomics studies, method standardization, open science workflows, and public metabolomics databases would largely benefit the tumor barcode construction and application.

## Data Availability

Not applicable.

## Supplementary Materials

The following supporting information can be downloaded at: https://www.mdpi.com/article/10.3390/metabo12060494/s1, Table S1: List of potential oncometabolites in blood sample; Table S2: List of potential oncometabolites in tissue samples; Table S3: Comparison of the performance of random forest and Xgboost to predict synergism; Figure S1: The Metabolite-protein network derived from 43 tissue oncometabolites; Figure S2: Tuning of *mtry* in random forest; Figure S3: Tuning of *max_depth* and *nround* (iterations) in the Xgboost model; Figure S4: ROC plots of random forest and Xgboost models in the synergy prediction; Figure S5: Determination of IC50 of CPT-11 on SW-620 (A) and HCT-116 (B) cell lines.
