# Supplementary material for: Discovery of Synergistic Drug Combinations for Colorectal Cancer Driven by Tumor Barcode Derived from Metabolomics “Big Data”
Source: Metabolites. 2022 May 30;12(6):494. doi: 10.3390/metabo12060494 (PMC9227693; doi:10.3390/metabo12060494)
Supplement: Supplementary file 1 [file metabolites-12-00494-s001.zip › metabolites-1693153-supplementary.pdf]

Table S1 List of potential oncometabolites in blood sample

Name	KEGG ID	Fre	Up	Down	Sum-Con	Sum-CRC	BF
Pyruvic acid	C00022	8	7	1	484	518	50.20
Acetic acid	C00033	4	4	0	205	266	31.00
L-Malic acid	C00149	3	3	0	125	154	15.00
2-Hydroxybutyric acid	C05984	3	3	0	187	186	15.00
L-Phenylalanine	C00079	8	6	2	1557	809	10.13
L-Isoleucine	C00407	5	4	1	389	367	8.14
Elaidic acid	C00712	5	4	1	217	272	8.14
L-Proline	C00148	7	5	2	403	448	5.92
3-Hydroxybutyric acid	C01089	7	5	2	265	389	5.92
Myoinositol	C00137	4	3	1	376	382	4.33
L-Arginine	C00062	4	3	1	1162	312	4.33
Fumaric acid	C00122	4	3	1	269	216	4.33
D-Fructose	C02336	4	3	1	399	374	4.33
L-Cystine	C00491	4	3	1	431	380	4.33
Glycine	C00037	6	4	2	389	400	3.41
L-Lactic acid	C00186	8	5	3	676	686	2.94
Pyroglutamic acid	C01879	3	2	1	115	118	2.20
D-Glutamic acid	C00217	3	2	1	177	150	2.20
Glycocholic acid	C01921	3	2	1	147	127	2.20
Glyceric acid	C00258	3	2	1	376	352	2.20
L-Glutamic acid	C00025	3	2	1	172	145	2.20
L-Aspartic acid	C00049	5	3	2	289	235	1.91
Glycerol	C00116	7	4	3	562	610	1.75
D-Glucose	C00031	4	2	2	226	192	1.00
Stearic acid	C01530	4	2	2	336	368	1.00
Linoleic acid	C01595	4	2	2	463	386	1.00
L-Asparagine	C00152	4	2	2	1137	340	1.00
Citric acid	C00158	6	3	3	500	548	1.00
Urea	C00086	4	2	2	293	335	1.00
L-Alanine	C00041	7	3	4	562	610	-1.75
Creatinine	C00791	5	2	3	289	235	-1.91
L-Methionine	C00073	5	2	3	1229	406	-1.91
Palmitic acid	C00249	3	1	2	316	322	-2.20

Name	KEGG ID	Fre	Up	Down	Sum-Con	Sum-CRC	BF
Oleamide	C19670	3	1	2	179	178	-2.20
4-Hydroxyproline	C01157	3	1	2	187	186	-2.20
Uric acid	C00366	3	1	2	207	154	-2.20
Ribitol	C00474	3	1	2	122	152	-2.20
Deoxyuridine	C00526	3	1	2	207	154	-2.20
L-Histidine	C00135	6	2	4	1284	434	-3.41
L-Serine	C00065	4	1	3	142	171	-4.33
L-Threonine	C00188	4	1	3	1120	353	-4.33
Citrulline	C00327	4	1	3	1175	351	-4.33
L-Tyrosine	C00082	7	2	5	1540	792	-5.92
L-Tryptophan	C00078	7	2	5	1257	462	-5.92
Ornithine	C00077	5	1	4	1202	404	-8.14
L-Leucine	C00123	6	1	5	1306	551	-15.00
L-Lysine	C00047	6	1	5	1521	647	-15.00
Creatine	C00300	3	0	3	186	228	-15.00
L-Valine	C00183	7	1	6	1306	581	-27.44
L-Glutamine	C00064	6	0	6	1333	553	-127.00

Fre represents frequency. *Up* represents numbers of studies reported the metabolite up-regulated. *Down* represents numbers of studies reported the metabolite down-regulated. *Sum-Con* represents cumulative control sample size, *Sum-CRC* represents cumulative CRC sample size. BF represents Bayesian Factors, in which positive (+) represents an up-regulation and negative (-) represents a down-regulation.

Table S2 List of potential oncometabolites in tissue samples

Name	KEGG ID	Fre	Up	Down	Sum-Con	Sum-CRC	BF
Taurine	C00245	8	8	0	202	202	511
L-Lactic acid	C00186	10	9	1	270	270	169.67
Hypoxanthine	C00262	6	6	0	123	123	127
L-Proline	C00148	6	6	0	137	137	127
L-Aspartic acid	C00049	6	6	0	195	195	127
L-Phenylalanine	C00079	5	5	0	170	170	63
Uracil	C00106	5	5	0	181	181	63
Glycine	C00037	8	7	1	219	219	50.2
Palmitic acid	C00249	4	4	0	83	83	31
L-Alanine	C00041	4	4	0	147	147	31
L-Leucine	C00123	4	4	0	122	122	31
L-Arginine	C00062	4	4	0	73	73	31
Glutathione	C00051	4	4	0	100	100	31
L-Cysteine	C00097	4	4	0	125	125	31
Choline	C00114	4	4	0	132	132	31
Carnitine	C00487	4	4	0	66	66	31
Stearic acid	C01530	3	3	0	74	74	15
L-Valine	C00183	3	3	0	113	113	15
L-Threonine	C00188	3	3	0	86	86	15
L-Isoleucine	C00407	3	3	0	113	113	15
D-Glutamic acid	C00217	3	3	0	116	116	15
L-Tyrosine	C00082	3	3	0	108	108	15
Phosphoric acid	C00009	3	3	0	96	96	15
Uridine	C00299	3	3	0	73	73	15
Myristic acid	C06424	3	3	0	74	74	15
Scyllitol	C06153	4	3	1	142	142	4.33
Adenine	C00147	4	3	1	61	61	4.33
L-Asparagine	C00152	4	3	1	133	133	4.33
Inosine	C00294	4	3	1	102	102	4.33
Glycerophosphocholine	C00670	3	2	1	111	111	2.2
L-Methionine	C00073	3	2	1	62	62	2.2

L-Alpha-aminobutyric acid	C02356	3	2	1	65	65	2.2
Succinic acid	C00042	3	2	1	67	67	2.2
Aminoadipic acid	C00956	3	2	1	71	71	2.2
Glycerol	C00116	3	2	1	74	74	2.2
Ornithine	C00077	4	2	2	85	85	1
Fumaric acid	C00122	3	1	2	78	78	-2.2
(R)-Malate	C00497	4	1	3	90	90	-4.33
Myoinositol	C00137	4	1	3	89	89	-4.33
D-Mannose	C00159	3	0	3	74	74	-15
Citric acid	C00158	3	0	3	36	36	-15
D-Galactose	C00124	7	1	6	154	154	-27.44
D-Glucose	C00031	7	0	7	164	164	-255

Fre represents frequency. *Up* represents numbers of studies reported the metabolite up-regulated. *Down* represents numbers of studies reported the metabolite down-regulated. *Sum-Con* represents cumulative control sample size, *Sum-CRC* represents cumulative CRC sample size. BF represents Bayesian Factors, in which positive (+) represents an up-regulation and negative (-) represents a down-regulation.

Table S3. Comparison of the performance of random forest and Xgboost in synergism prediction

	Random Forest	Xgboost
Specificity	0.887	0.839
Sensitivity	0.857	0.905
True positive rate	0.837	0.792
True negative rate	0.902	0.929
Accuracy in 10-fold cross validation	0.875	0.865
Accuracy in test set	0.845	0.808
AUC of ROC	0.9067-0.9869	0.9292-0.9905

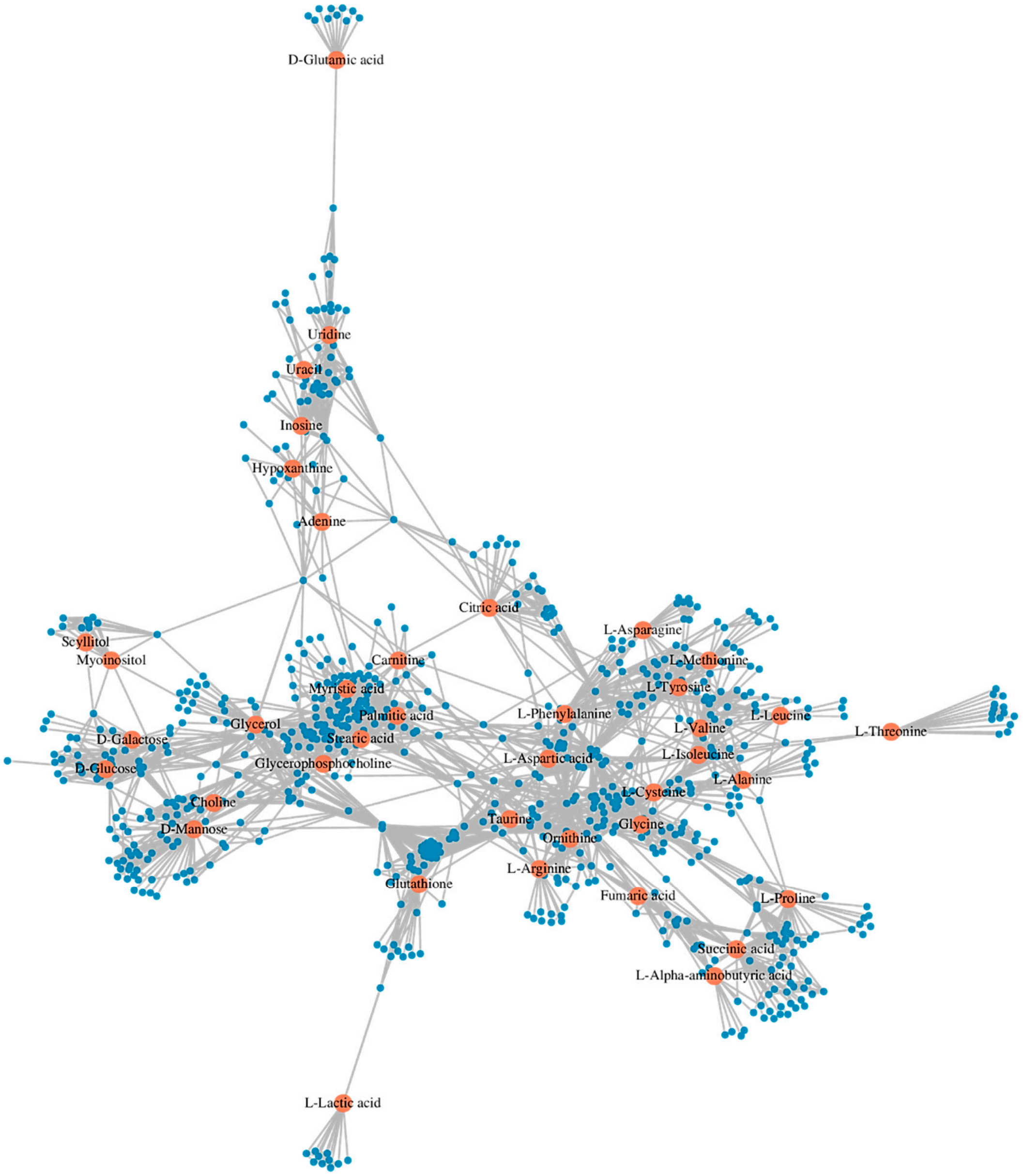

Figure S1. The Metabolite-protein network derived from 43 tissue oncometabolites.

Note: isolated islands were removed. In total 804 nodes and 9403 interactions are presented.

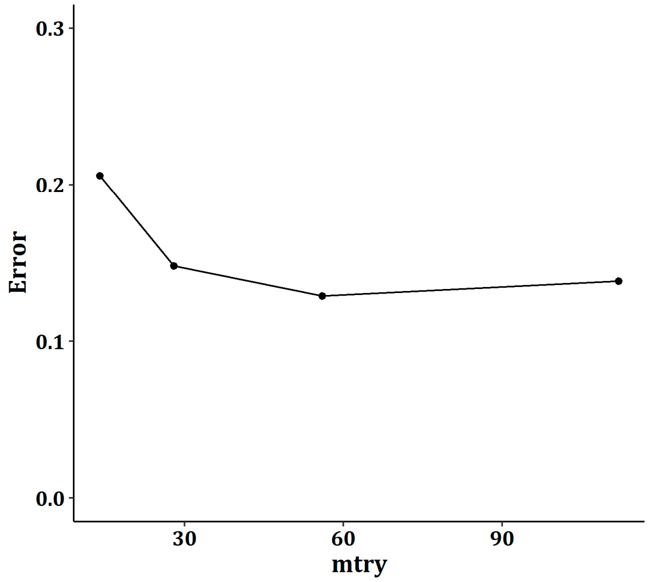

Figure S2. Tuning of the parameter of *mtry* in random forest.

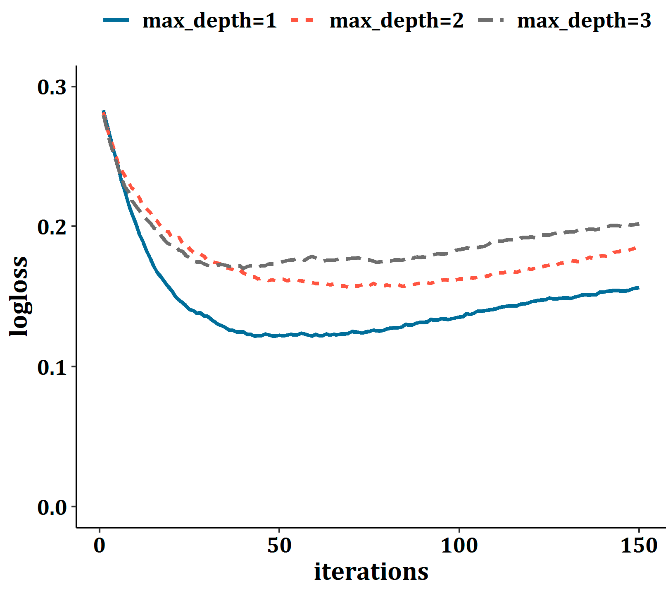

Figure S3. Tuning of *max_depth* and *nround* (iterations) in the Xgboost model.

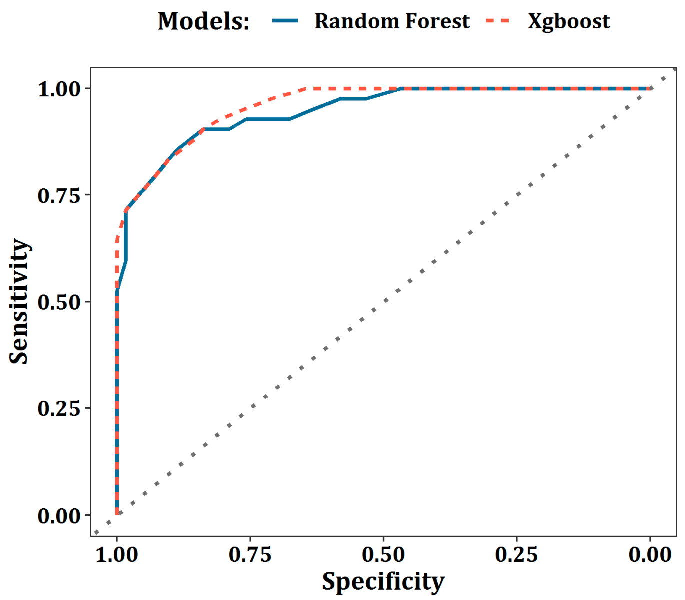

Figure S4. ROC plots of random forest and Xgboost models in the synergy prediction.

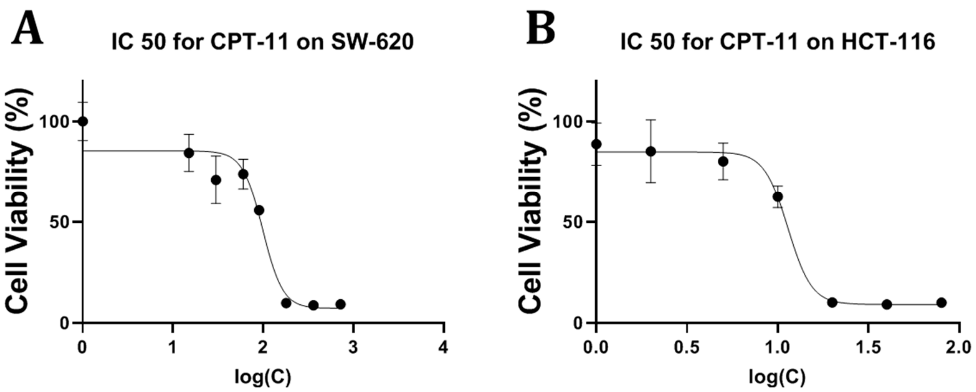

Figure S5. Determination of IC_{50} of CPT-11 on SW-620 (A) and HCT-116 (B) cell lines.
